# A Rare and Aggressive Klatskin Tumor Revealed by Magnetic Resonance Cholangiopancreatography (MRCP): A Diagnostic Case Report

**DOI:** 10.7759/cureus.100743

**Published:** 2026-01-04

**Authors:** Edgar A Flores García, Axell D Lugo Rodríguez, Jennifer Navarro Morales, José I Rodríguez Murua, Juan F Maciel, Jorge A Favela Ramos, Jorge A Vazquez Tovar

**Affiliations:** 1 General Surgery, Hospital General Nuevo Gómez Palacio, Gómez Palacio, MEX; 2 General Surgery, Instituto Mexicano del Seguro Social, Torreón, MEX; 3 General Surgery, Hospital Nuevo Gómez Palacio, Gómez Palacio, MEX; 4 Internal Medicine, Hospital Nuevo Gómez Palacio, Gómez Palacio, MEX; 5 General Surgery, Hospital Nuevo Gómez Palacio, Gomez Palacio, MEX

**Keywords:** classification bismuth- corlette, hilar cholangiocarcinoma, klatskin tumor, magnetic resonance cholangiopancreatography (mrcp), perihilar cholangiocarcinoma

## Abstract

A 57-year-old man with controlled hypertension and moderate daily alcohol and tobacco consumption presented with one week of progressive severe obstructive jaundice, intense pruritus, acholic stools, and dark urine. Total bilirubin on admission was 25.1 mg/dL, rising to 29.4 mg/dL within 48 hours despite supportive care. Initial endoscopic retrograde cholangiopancreatography (ERCP) completely failed due to the inability to cannulate the biliary tree. Tumor markers obtained after this failure revealed a strikingly elevated CA 19-9 of 4,872 U/mL and carcinoembryonic antigen (CEA) of 18.6 ng/mL. Magnetic resonance cholangiopancreatography (MRCP) demonstrated an infiltrative stricture at the hepatic duct confluence with abrupt ductal termination, separation of right and left hepatic ducts, a shouldering sign, and predominant right-lobe intrahepatic biliary dilatation - findings diagnostic of Bismuth-Corlette type IIIa Klatskin tumor. No vascular encasement or distant metastases were identified. A second ERCP successfully deployed an uncovered 10 × 80 mm self-expanding metal stent, leading to rapid clinical and biochemical improvement. The patient was promptly referred to a specialized hepatobiliary unit for evaluation of curative-intent resection. This case highlights how contemporary high-resolution MRCP combined with extreme CA 19-9 elevation can establish a confident noninvasive diagnosis of Klatskin tumor when initial ERCP fails, dramatically shortening diagnostic delay and accelerating the pathway to potentially curative surgery.

## Introduction

Hilar cholangiocarcinoma, also termed Klatskin tumor, constitutes 50-70% of all cholangiocarcinomas and originates from the biliary epithelium at or immediately adjacent to the hepatic duct confluence [1,2]. It accounts for approximately 10% of primary hepatobiliary malignancies, with an age-adjusted incidence of one to two per 100,000 population in Western countries; however, its incidence appears to be increasing worldwide [1-3]. Classic risk factors include primary sclerosing cholangitis, choledochal cysts, chronic viral hepatitis, and hepatolithiasis, although many patients lack identifiable predisposing conditions [2,3].

The Bismuth-Corlette classification remains the most widely used system for preoperative anatomic staging: Type I involves the common hepatic duct below the confluence; Type II reaches the confluence without involvement of secondary ducts; Type IIIa and IIIb extend into the right or left secondary intrahepatic ducts, respectively; and Type IV demonstrates bilateral secondary duct involvement or multifocal disease [3]. This classification directly influences resectability, with Types I-IIIa generally amenable to curative-intent resection (often requiring major hepatectomy) and Type IV usually precluding R0 resection [4,5]. R0 resection is defined as complete tumor removal with histologically negative surgical margins and no microscopic evidence of residual disease.

Accurate preoperative diagnosis and staging are challenging due to the tumor’s predominant periductal-infiltrating growth pattern, marked desmoplastic reaction, and frequent absence of a discrete mass on cross-sectional imaging [1,6]. Tissue acquisition via endoscopic or percutaneous routes carries low diagnostic yield (30-50%) and risks of seeding or infectious complications [1,6]. Magnetic resonance cholangiopancreatography (MRCP), particularly when combined with contrast-enhanced sequences and diffusion-weighted imaging, has become the cornerstone non-invasive diagnostic modality, achieving sensitivity and specificity exceeding 90% for detecting malignant hilar strictures and accurately delineating longitudinal ductal involvement [7,8]. MRCP also facilitates assessment of vascular encasement, lobar atrophy, and distant metastases - critical determinants of resectability - often obviating the need for diagnostic ERCP [4,7].

## Case presentation

A 57-year-old male presented to the emergency department with a one-week history of progressive jaundice, intense pruritus, clay-colored stools, and dark urine. He denied abdominal pain, fever, weight loss, or other associated symptoms. Past medical history was notable for arterial hypertension controlled with enalapril. Social history included daily consumption of two to three beers and two cigarettes per day. He denied diabetes mellitus, prior transfusions, surgeries, or known allergies.

Physical examination revealed marked scleral and cutaneous icterus without abdominal tenderness, palpable masses, or hepatosplenomegaly. Initial laboratory evaluation showed total bilirubin 25.1 mg/dL (direct 19.8 mg/dL), alkaline phosphatase 812 IU/L, gamma-glutamyl transferase 1,124 IU/L, asparatate aminotransferase (AST) 118 IU/L, and ALT 212 IU/L (Table 1). Abdominal ultrasound was nondiagnostic due to overlying bowel gas.

**Table 1:** Liver function test results in the present case compared with laboratory reference ranges.

Measurement	Normal Range	Patient Value
Total Protein	6.3-8.2 gr/dl	6.2 g/dl
Albumin	3.5-5.0 gr/dl	3 g/dl
Globulin	2.8-3.2 gr/dl	3.2 g/dl
Albumin/Globulin (A/G) Ratio	1.1-2.2 gr/dl	0.9 g/dl
Aspartate Aminotransferase (AST)	10-40 u/l	118 u/l
Alanine Aminotransferase (ALT)	10-41 u/l	212 u/l
Gamma-Glutamyl Transferase (GGT)	5 - 40 u/l	1,124 u/l
Alkaline Phosphatase	38 -126 u/l	812 u/l
Total Bilirubin	0.2-1.3 mg/dl	25.1 mg/dl
Direct Bilirubin	0-0.4 mg/dl	19.8 mg/dl
Indirect Bilirubin	0-0.6 mg/dl	2.7 mg/dl

A first endoscopic retrograde cholangiopancreatography (ERCP) was attempted, but selective biliary cannulation failed, precluding diagnostic brushings or stent placement. Following this unsuccessful procedure, serum tumor markers were obtained, revealing CA 19-9 of 4,872 U/mL (reference <37 U/mL) and carcinoembryonic antigen (CEA) of 18.6 ng/mL (reference <5 ng/mL) (Table 2). Repeat laboratories 48 hours later demonstrated worsening hyperbilirubinemia (total bilirubin 29.4 mg/dL).

**Table 2:** Tumor marker levels (CA 19-9 and CEA) in the present case compared with laboratory reference ranges. CA 19-9 = carbohydrate antigen 19-9

Measurement	Normal Range	Patient Value
CA 19-9 Antigen	0-36 u/ml	4,872 u/ml
Carcinoembryonic Antigen (CEA)	0-5.0 ng/ml	18.6 ng/ml

**Figure 1:**
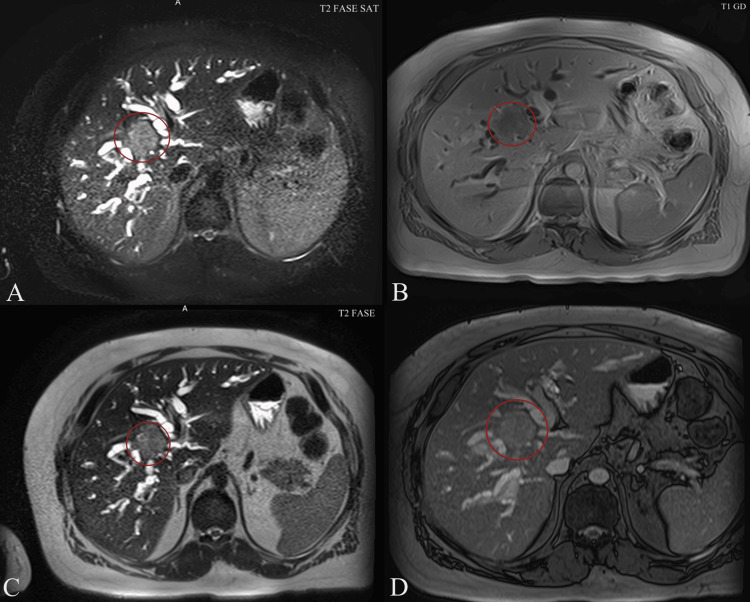
Magnetic resonance cholangiopancreatography (MRCP) and multiphasic MRI (axial planes) in a patient with Bismuth-Corlette type IIIa perihilar cholangiocarcinoma (Klatskin tumor). A) T2-weighted fat-suppressed sequence showing marked intrahepatic biliary dilatation with abrupt termination at the hepatic hilum (red circle). (B) T1-weighted gadolinium-enhanced arterial phase demonstrating mild heterogeneous enhancement of the hilar mass (red circle). (C) Standard T2-weighted sequence revealing bilateral intrahepatic duct dilatation and the “peripheral duct sign” (red circle). (D) T1-weighted gadolinium-enhanced delayed phase showing progressive delayed enhancement of the hilar tumor with adjacent capsular retraction (red circle), a finding highly suggestive of perihilar cholangiocarcinoma. Tumor classification is based on the Bismuth–Corlette classification system as originally described by Bismuth et al. (1992) [3].

**Figure 2:**
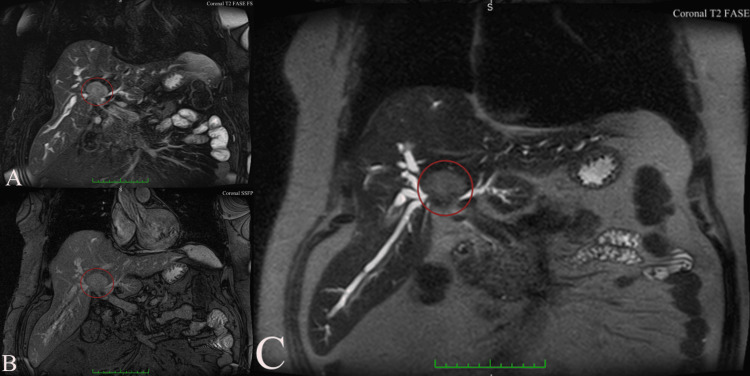
Coronal MR imaging sequences from the same patient with Bismuth-Corlette type IIIa perihilar cholangiocarcinoma. (A) T2-weighted fat-suppressed coronal image demonstrating marked bilateral intrahepatic biliary dilatation with abrupt cutoff at the hepatic hilum (red circle). (B) Coronal steady-state free precession (SSFP/TrueFISP) sequence providing an overview of the hilar stricture with complete separation of the right and left hepatic ducts (red circle). (C) Thick-slab heavily T2-weighted coronal MRCP image showing high-grade stricture of the biliary confluence with symmetric upstream dilatation of the right and left ductal systems, classic appearance of a Bismuth-Corlette type IIIa Klatskin tumor (red circle)[3]. Tumor classification is based on the Bismuth–Corlette classification system as originally described by Bismuth et al. (1992) [3]. TrueFISP: True Fast Imaging with Steady state Precession (Siemens Healthineers, Erlangen, Germany); MRCP: Magnetic resonance cholangiopancreatography

**Figure 3:**
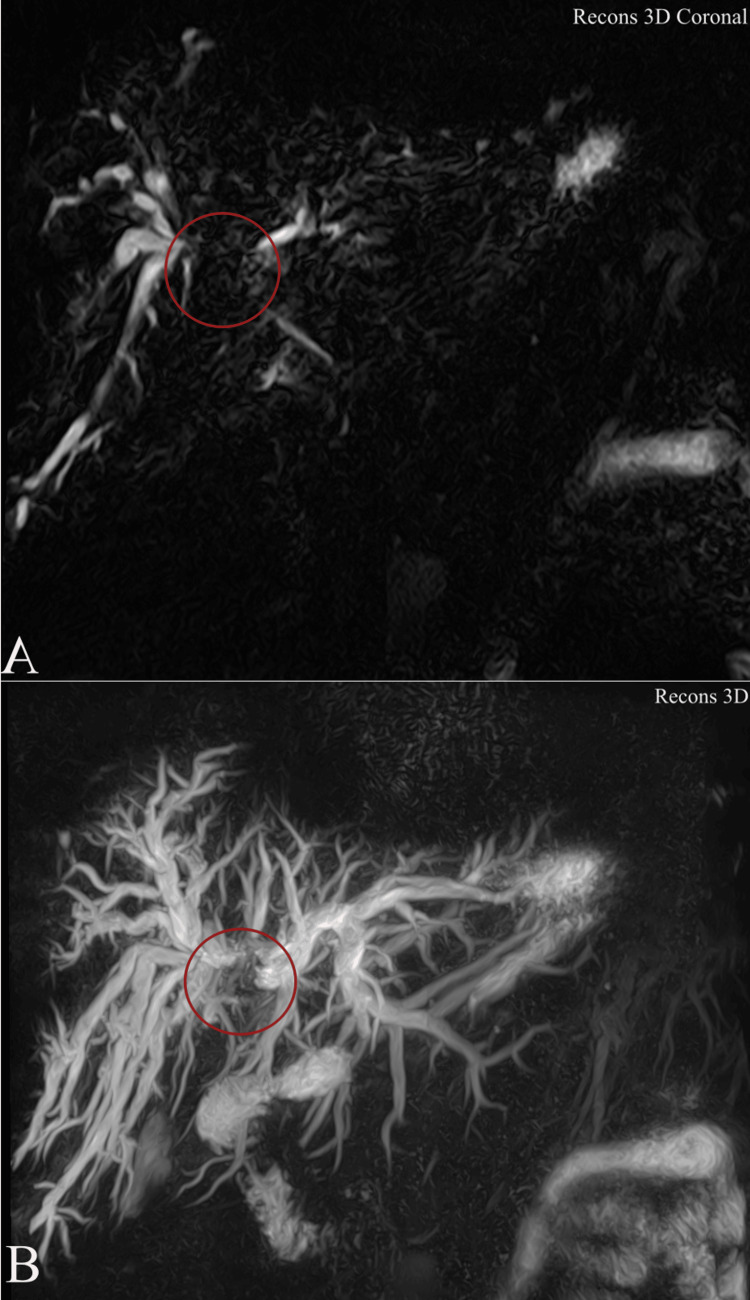
Three-dimensional reconstructions from magnetic resonance cholangiopancreatography in the same patient with Bismuth-Corlette type IIIa perihilar cholangiocarcinoma. (A) Anterior coronal 3D heavily T2-weighted MIP reconstruction showing bilateral intrahepatic biliary dilatation with complete obliteration of the biliary confluence and >1 cm separation of the proximal right and left hepatic ducts (red circle). (B) Slightly oblique inferior 3D view confirming tumor involvement of the proximal right hepatic duct while the left biliary system remains patent. Tumor classification is based on the Bismuth–Corlette classification system as originally described by Bismuth et al. (1992) [3]. MIP: Maximum Intensity Projection

Magnetic resonance cholangiopancreatography (MRCP) demonstrated an infiltrative hilar mass with irregular ductal stricturing and abrupt termination of the right and left hepatic ducts at the confluence, consistent with Klatskin tumor (Bismuth-Corlette type IIIa) (Figure 1) [3]. Proximal intrahepatic biliary ducts were markedly dilated, predominantly on the right side (Figure 2). Contrast-enhanced sequences showed wall enhancement of the involved ducts without definite portal vein or hepatic artery encasement or distant metastases (Figure 3).

A second ERCP was successfully performed two days later, during which a 10 mm × 80 mm uncovered self-expanding metal stent was deployed across the stricture, achieving adequate biliary drainage. Post-procedure bilirubin levels progressively declined.

The patient was subsequently referred to the hepatobiliary surgery and medical oncology services for multidisciplinary evaluation and consideration of curative-intent resection.

Notably, the serum CA 19-9 level was extraordinarily elevated at 4,872 U/mL (reference <37 u/ml), a value more than 130 times the upper limit of normal. This markedly high titer, observed early in the diagnostic workup following the unsuccessful initial ERCP, immediately raised strong suspicion for hilar cholangiocarcinoma, even in the absence of initial cytological confirmation, and prompted expedited advanced imaging with MRCP to delineate the extent of disease.

## Discussion

Hilar cholangiocarcinoma (Klatskin tumor) accounts for approximately two-thirds of all cholangiocarcinomas and continues to pose significant diagnostic and therapeutic challenges due to its location, periductal growth pattern, and delayed clinical presentation [1,2]. The present case exemplifies several key aspects of contemporary management: the limitations of endoscopic retrograde cholangiopancreatography (ERCP) as a first-line procedure in complex hilar strictures, the central role of magnetic resonance cholangiopancreatography (MRCP) in establishing a confident noninvasive diagnosis, and the prognostic implications of markedly elevated serum CA 19-9.

**Figure 4:**
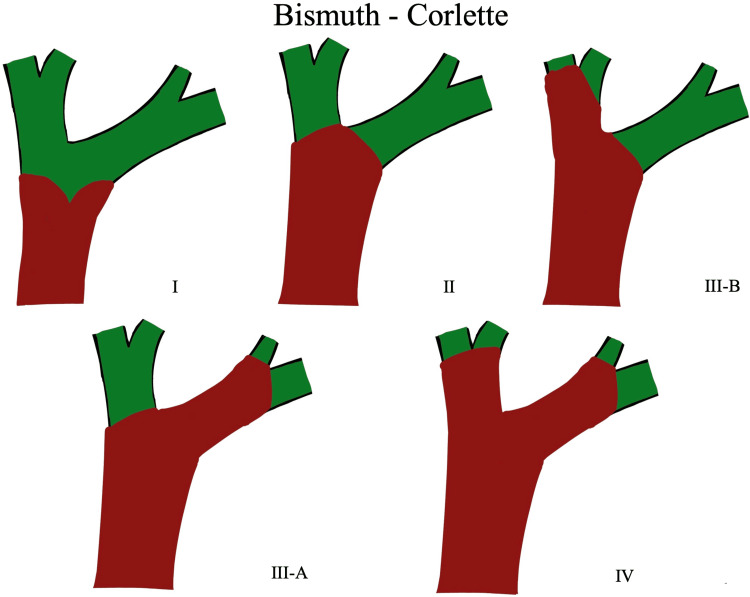
Bismuth-Corlette classification of perihilar cholangiocarcinoma: schematic representation of tumor extension along the biliary confluence Type I: Tumor confined to the common hepatic duct, below the confluence of the right and left hepatic ducts. Type II: Tumor reaches the biliary confluence without extension into the right or left hepatic ducts. Type IIIa: Tumor extends into the right hepatic duct. Type IIIb: Tumor extends into the left hepatic duct. Type IV: Tumor involves both right and left hepatic ducts and/or multifocal disease with extension to second-order biliary radicals. Created by the authors for educational purposes based on the general principles of the Bismuth–Corlette classification [3].

Obstructive jaundice with acholic stools, choluria, and pruritus represents the most common initial manifestation, occurring in >90% of patients [1,2]. The rapid rise in serum bilirubin to 29.4 mg/dL observed in our patient reflects the complete or near-complete hilar obstruction typical of Bismuth-Corlette type III lesions (Figure 4) [3,4]. Failure of biliary cannulation during the initial ERCP, a recognized complication in 10-20% of malignant hilar strictures, precluded both cytologic sampling and immediate decompression [6,8]. Such technical difficulties have prompted a paradigm shift toward earlier reliance on cross-sectional imaging rather than diagnostic ERCP [4,7].

Magnetic resonance cholangiopancreatography, particularly when performed with 3D sequences and diffusion-weighted imaging, currently achieves diagnostic accuracy of 90-96% for malignant hilar obstruction and accurately delineates ductal and vascular involvement in >85% of cases [7,8]. The characteristic findings in this patient - abrupt termination of the hepatic ducts at the confluence, irregular wall thickening with shouldering, and asymmetric intrahepatic dilatation - permitted a definitive radiologic diagnosis of Bismuth-Corlette type IIIa Klatskin tumor without histologic confirmation, an approach endorsed by expert consensus when imaging is unequivocal [4,5]. It should be noted that the reported diagnostic accuracy of MRCP exceeding 90-96% primarily refers to the differentiation between malignant and benign biliary strictures, rather than to precise histologic tumor typing or definitive distinction among specific biliary malignancies [7,8]. The concomitant CA 19-9 level of 4,872 U/mL substantially exceeded reported cutoffs associated with malignancy (>1,000 U/mL in the setting of jaundice) and strengthened diagnostic certainty despite the known influence of biliary obstruction on marker elevation [1,2].

Successful placement of an uncovered self-expanding metal stent during repeat ERCP achieved effective biliary drainage, consistent with technical success rates of 85-95% and clinical response in >80% of patients with malignant hilar obstruction [8,9]. Absence of definite vascular encasement or distant metastases on MRCP suggests potential resectability, with reported R0 resection rates of 56-78% for type IIIa lesions at high-volume centers when appropriate preoperative biliary decompression and portal vein embolization are employed [10,11].

This case highlights the evolving diagnostic algorithm for suspected Klatskin tumor, in which high-resolution MRCP, supported by significantly elevated tumor markers, can obviate the need for tissue diagnosis in selected patients and expedite referral for potentially curative resection [12,13]. It also underscores the importance of early involvement of multidisciplinary hepatobiliary teams to optimize outcomes in this aggressive malignancy with a historically poor prognosis [14,15].

In the present case, although magnetic resonance cholangiopancreatography (MRCP) is currently the noninvasive modality of choice for evaluating the biliary tree in patients with suspected hilar cholangiocarcinoma due to its high resolution and lack of ionizing radiation, it was not performed initially for several reasons. First, the patient presented with acute obstructive jaundice and signs of cholangitis, necessitating urgent biliary decompression via diagnostic and therapeutic ERCP. ERCP not only allowed direct cytological and histological sampling but also immediately resolved biliary sepsis, thereby prioritizing clinical stabilization over a purely diagnostic imaging study. Additionally, at the time of admission, institutional availability of MRCP was limited during off-hours, which influenced the diagnostic sequencing. In patients with a similar presentation - acute jaundice and suspected infection - we consider it reasonable to prioritize procedures that combine diagnosis and treatment, reserving MRCP for preoperative staging once the acute phase has been controlled, as was subsequently performed in this case.

Given that this is a unique clinical case, the observations described herein should not be generalized to all patients with hilar cholangiocarcinoma; prospective studies are required to validate these findings.

## Conclusions

This case demonstrates the current diagnostic paradigm shift in suspected Klatskin tumor when initial ERCP fails. High-resolution magnetic resonance cholangiopancreatography (MRCP), particularly when combined with markedly elevated serum CA 19-9 (>4,000 U/mL in the setting of jaundice), can provide a confident noninvasive diagnosis of Bismuth-Corlette type IIIa hilar cholangiocarcinoma without the need for tissue confirmation. This approach avoids the risks and delays associated with repeated unsuccessful endoscopic or percutaneous procedures.

Prompt recognition of characteristic MRCP findings - abrupt ductal separation, shouldering sign, asymmetric intrahepatic dilatation, and wall enhancement - allowed rapid biliary decompression via self-expanding metal stent placement and immediate referral to a specialized hepatobiliary unit while the patient remained a potential candidate for curative-intent resection. In an era of evolving multimodal therapy, early noninvasive diagnostic certainty is critical to optimize outcomes in this aggressive malignancy with a historically dismal prognosis.
